# Inhibition of RNA polymerase III transcription by Triptolide attenuates colorectal tumorigenesis

**DOI:** 10.1186/s13046-019-1232-x

**Published:** 2019-05-23

**Authors:** Xia Liang, Renxiang Xie, Jinfeng Su, Bingqi Ye, Saisai Wei, Zhibing Liang, Rongpan Bai, Zhanghui Chen, Zhongxiang Li, Xiangwei Gao

**Affiliations:** 1 Medical Research Institute, & Guangdong Women and Children’s Disease Precision Diagnosis and Treatment Engineering Technology Research Center, Baoan Maternal and Child Health Hospital, Jinan University, Shenzhen, 518102 China; 2 Department of Public Health, Zhejiang University School of Medicine, Hangzhou, 310058 China; 3 Sir Run-Run Shaw Hospital, Zhejiang University School of Medicine, Hangzhou, 310058 China; 4 Affiliated Central People’s Hospital of Zhanjiang, Guangdong Medical University, Zhanjiang, 524045 China

**Keywords:** Triptolide, Colorectal cancer, RNA polymerase III, TFIIIB

## Abstract

**Background:**

Upregulation of RNA polymerase (Pol) III products, including tRNAs and 5S rRNA, in tumor cells leads to enhanced protein synthesis and tumor formation, making it a potential target for cancer treatment. In this study, we evaluated the inhibition of Pol III transcription by triptolide and the anti-cancer effect of this drug in colorectal tumorigenesis.

**Methods:**

The effect of triptolide on colorectal cancer development was assessed in colorectal cancer mouse models, 3D organoids, and cultured cells. Colorectal cancer cells were treated with triptolide. Pol III transcription was measured by real-time quantitative polymerase chain reaction (PCR). The formation of TFIIIB, a multi-subunit transcription factor for Pol III, was determined by chromatin immunoprecipitation (ChIP), co-immunoprecipitation (Co-IP), and fluorescence resonance energy transfer (FRET).

**Results:**

Triptolide reduced both tumor number and tumor size in adenomatous polyposis coli (*Apc*) mutated (Apc^Min/+^) mice as well as AOM/DSS-induced mice. Moreover, triptolide effectively inhibited colorectal cancer cell proliferation, colony formation, and organoid growth in vitro, which was associated with decreased Pol III target genes. Mechanistically, triptolide treatment blocked TBP/Brf1interaction, leading to the reduced formation of TFIIIB at the promoters of tRNAs and 5S rRNA.

**Conclusions:**

Together, our data suggest that inhibition of Pol III transcription with existing drugs such as triptolide provides a new avenue for developing novel therapies for colorectal cancer.

**Electronic supplementary material:**

The online version of this article (10.1186/s13046-019-1232-x) contains supplementary material, which is available to authorized users.

## Background

Colorectal cancer (CRC) is the third most common cause of cancer-related deaths worldwide [1]. Despite significant progress in the prevention, early diagnosis and management of CRC, the clinical outcome for advanced CRC is unsatisfactory [1]. Chemotherapy compounds such as 5-FU and oxaliplatin are well established in reducing recurrence and prolonging survival, especially for later stage patients. However, the systemic toxicity caused by chemotherapy and the development of drug resistance are major obstacles for the success of CRC chemotherapy [2]. Thus, the development of novel anticancer drugs or therapeutics is urgently needed.

Triptolide is a diterpene triepoxide purified from Tripterygium wilfordii Hook F, commonly known as lei gong teng or thunder god vine, a medicinal plant whose extracts have been used in traditional Chinese medicine for treating rheumatoid arthritis and other inflammatory diseases [3]. Recent studies have shown that triptolide kills a variety of cancer cell lines including colorectal cancer cells in vitro with high potency [4]. Animal studies have shown that triptolide inhibits the growth of colorectal cancer cells in a mouse xenograft model [5]. However, cell lines do not reflect the complexity of in vivo tumor growth condition, while xenograft model may not represent the real context of colorectal carcinogenesis. Apc^Min/+^ mouse, carrying a truncation mutation at the tumor suppressor gene *Apc*, is a frequently used genetic mouse model for the study of familial adenomatous polyposis (FAP) and early events of CRC [6]. Genotoxic colonic carcinogen such as azoxymethane (AOM) is routinely used to induce colon tumors in mice [7]. The combination of AOM and dextran sodium sulfate (DSS) is frequently used to investigate the development of colitis-associated cancer (CAC) [8]. Therefore, evaluation of the anti-cancer effects of triptolide using these colorectal carcinogenesis mouse models will promote its clinical applications for CRC treatment.

The anticancer effect of triptolide was attributed to its inhibition on transcription [9]. It was reported that triptolide targets the transcription factor XPB and RNA polymerase (Pol) II to inhibit Pol II-mediated mRNA transcription [10, 11]. XPB, a subunit of the general transcription factor TFIIH, is involved in both RNAPII-mediated transcription initiation and DNA nucleotide excision repair. Triptolide directly binds to XPB, inhibits its ATPase activity and consequently, represses RNAPII transcription initiation and nucleotide excision repair [10]. On the other hand, triptolide induces the degradation of Rpb1, the largest subunit of RNA polymerase II [11]. It was also reported that triptolide disrupts nucleolar integrity and inhibits Pol I-mediated ribosomal RNA transcription [12]. Therefore, triptolide acts as an inhibitor of both Pol I and Pol II.

Pol III, the third type of RNA polymerase, transcribes a variety of untranslated RNAs, including tRNAs, 5S rRNA, 7SL RNA, 7SK RNA and U6 RNA [13], whereas tRNAs and 5S rRNA control mRNA translation efficiency and growth capacity of cells [14, 15]. Polymerase III transcription is mediated by TFIIIB complex, consisting of TATA box-binding protein (TBP) and its associated factors B-related factor 1 (Brf1), and B double prime 1 (Bdp1). TFIIIB, together with TFIIIC and RNA Pol III, mediate tRNAs transcription, whereas TFIIIB, together with TFIIIA, TFIIIC and RNA Pol III, mediate 5S rRNA genes transcription [16]. Studies have indicated that RNA Pol III products are elevated in both transformed and tumor cells, suggesting that they have a crucial role in tumorigenesis [14, 17]. Consistent with this idea, enhanced Pol III transcription is required for oncogenic transformation [18]. However, whether triptolide could target Pol III transcription remains to be identified.

In this study, we systematically evaluated triptolide as a therapeutic option for the treatment of colorectal cancer in both Apc^Min/+^ and AOM/DSS mice. Additionally, our data revealed the inhibitory effect of triptolide on Pol III transcription, which has important implications in the application of triptolide for CRC treatment.

## Materials and methods

### Mouse treatment

To evaluate the effect of triptolide on tumorigenesis in vivo, both AOM/DSS and Apc^Min/+^ mouse CRC models were used. For the AOM/DSS model, eight-week-old wild type female C57BL/6 J mice were first injected with 10 mg/kg AOM intraperitoneally 1 week prior to the first of three DSS cycles. After the formation of tumors (usually 8 weeks after AOM treatment), mice were randomly divided into two groups and intraperitoneal administrated with either 0.5 mg/kg of triptolide in dimethyl sulfoxide (DMSO) or DMSO (solvent only, control group) twice a week. The mice were sacrificed at 12-week of age and the number of tumors was assessed. The diameter of the tumors was measured and categorized them to 3 groups: small (< 2 mm), middle (2–4 mm), and large (> 4 mm). Six mice were used for each group.

The Apc^Min/+^ mice, carrying a truncated mutation of the Apc gene, develop spontaneous intestinal tumors [6]. After the formation of tumors (usually happens at 3 months of age), the Apc^Min/+^ mice were randomly divided into two groups and treated with either 0.5 mg/kg of triptolide in DMSO or DMSO (solvent only, control group) twice a week. The mice were sacrificed at six-month of age and the number, size of tumors was assessed. Seven mice were used for each group.

Mice were maintained and bred in specific pathogen-free conditions at the Animal Center of Zhejiang University. All animal studies were performed in compliance with the Guide for the Care and Use of Laboratory Animals by the Medical Experimental Animal Care Commission of Zhejiang University. All animal studies used the protocol that has been approved by the Medical Experimental Animal Care Commission of Zhejiang University.

### Organoid culture and ATP measurement

The crypt was isolated from the Apc^Min/+^ mouse intestine using ethylenediaminetetraacetic acid (EDTA). A total of 500 isolated crypts were mixed with 50 μL of Matrigel (BD Bioscience, San Jose, CA, United States) and plated in 24-well plates. After matrigel polymerization, 500 μL of IntestiCult Organoid Growth Medium (STEMCELL Technologies, Vancouver, Canada) was added on the top for organoid culture. Organoids were maintained at 37 °C in a Forma incubator (Thermo Fisher Scientific, Waltham, MA, United States) containing 5% CO2 and 95% humidity. Organoids were treated with different concentrations of triptolide for 72 h. ATP levels were measured using the Cell-Titer Glo2.0 Cell Viability Assay Kit (Promega, Madison, WI, United States) according to the manufacturer’s instructions.

### Cell culture and treatment

Colorectal cancer cells RKO and HCT116 were obtained from ATCC and cultured in DMEM medium supplemented with 10% fetal bovine serum (Thermo Fisher Scientific). Cells were maintained at 37 °C in a Forma incubator (Thermo Fisher Scientific) containing 5% CO_2_ and 95% humidity. Cells seeded in plates were treated with triptolide at the different concentration for the indicated time and then subjected to further experiments.

### CCK8-based viability assay

The effect of Triptolide on cell viability was assessed by Cell Counting Kit-8 (CCK8) assay (Dojindo Laboratory, Japan). RKO cells or HCT116 cells were then treated with Triptolide for 48 h. 10 μl of CCK8 reagent was added to each well and the cells were incubated for 2 h at 37 °C. The optical density (OD) at 450 nm was measured by using *Varioskan*Flash (Thermo Scientific).

### Cell cycle analysis

The effect of triptolide on cell cycle was assessed using propidium iodide staining and flow cytometry. RKO cells or HCT116 cells treated with or without triptolide at different concentrations for 24 h were fixed with 70% ice-cold ethanol for 12 h, stained with propidium iodide (5 μM, BioVision, Inc.) for 30 min at room temperature and examined with a flow cytometer as mentioned before (Beckman Coulter, Brea, CA, United States). DNA histograms were analyzed.

### Colony formation assay

To detect the effect of triptolide on colony formation, cells were seeded at a density of 500 cells/well in a 6-well plate and allowed to adhere for 24 h. The cells were then treated with triptolide of 5, 10 nM or control DMSO (designated as 0 nM). After 24 h, the medium was replaced with fresh medium and replaced again every 3 days thereafter. The cells were grown for 10 days, then fixed using methanol and stained with 1% crystal violet. Wells containing more than 50 cells were counted. The experiment was performed at least three times. The number of colonies in triptolide group was analyzed by comparing with DMSO control.

### RNA purification and reverse transcription reaction

Total RNA was isolated with Trizol reagent (Life Technologies, Grand Island, NY, United States) following the manufacturer’s protocol. 0.5 μg of total RNA was reverse transcribed using random hexamers and the High Capacity cDNA Reverse Transcription Kit (Life Technologies).

### The real-time quantitative PCR analysis

The real-time quantitative PCR analysis was performed in 10-μl reactions using SYBR GREEN PCR Master Mix (Applied Biosystems). The related mRNA level was normalized to the *β-actin* mRNA level. Data were analyzed using the 2^−ΔΔCt^ method [19]. Sequences of all the primers used for PCR amplification are listed in Additional file 1: Table S1.

### Immunoblotting analysis

Proteins were quantified by BCA protein assay kit (Beyotime Biotechnology, Shanghai, China) and applied to immunoblotting analysis as described previously [20]. 50 μg of total proteins were subjected to sodium dodecyl sulfate polyacrylamide gel electrophoresis (SDS-PAGE) and transferred to nitrocellulose membrane (Whatman, Clifton, NJ, United States). Membrane was blocked with 3% bovine serum albumin in TBS-T buffer (20 mM Tris-HCl, pH 8.0, 150 mM NaCl, 0.05% Tween- 20), probed with antibodies targeting to FLAG-tag (Cell Signaling Technology, Beverly, MA, United States), Myc-tag (Cell Signaling Technology), TBP (Cell Signaling Technology), POLR3D (Abcam, Cambridge, MA, United States), or ACTB (Cell Signaling Technology). The membrane was incubated with horseradish-conjugated secondary antibodies and visualized using enhanced chemiluminescence.

### Puromycin labeling

Cells at 70–80% confluence were treated with 10 μg/mL puromycin for 10 min. After washing with ice-cold PBS, cells were lysed and proteins were subjected to immunoblotting with puromycin antibody.

### Chromatin immunoprecipitation (ChIP)

ChIP assays were performed using the ChIP assay kit (Thermo Fisher Scientific) following the manufacturer’s protocol. Briefly, RKO cells transfected with FLAG-tagged proteins were cross-linked with 1% formaldehyde for 10 min at 37 °C. Cross-linking was stopped with 0.125 M glycine. After sonication to yield DNA fragments of 300–1000 base pairs, the lysates were cleared by centrifugation, diluted 6-fold with ChIP dilution buffer, and precleared with salmon sperm DNA/protein A-agarose at 4 °C for 1 h. For each immunoprecipitation assay, the lysates were incubated with 2 μg of anti-FLAG (Sigma) or control IgGs (Santa Cruz Biotechnology) overnight at 4 °C with rotation. The immunocomplexes were then collected with protein A-agarose slurry, eluted, and de-crosslinked at 65 °C. After RNase digestion and proteinase digestion, immunoprecipitated DNA was extracted. The purified DNA was amplified by real-time PCR.

Sequences of the primers used for tRNA^Leu^ and 5S rRNA promoter amplification are listed in Additional file 1: Table S1.

### Plasmids

For the construction of FLAG-tagged Brf1, TBP, or POLR3D, the corresponding genes were amplified by PCR and cloned to the vector p3XFLAG-CMV-13 between HindIII and EcoRI sites. To construct myc-tagged Brf1, Brf1 gene was amplified and cloned to the vector pcDNA3.1-myc-his B between HindIII and EcoRI sites. TBP and POLR3D genes were cut by HindIII/KpnI from p3XFLAG-CMV-13 and subcloned to pEYFP-N1 to get YFP-tagged fusion genes. Brf1 gene was cut by HindIII/KpnI from p3XFLAG-CMV-13 and subcloned to pECFP-N1 to get CFP-tagged fusion gene.

All the oligo sequences were listed in Additional file 1: Table S1.

### Co-immunoprecipitation (co-IP)

Co-IP was carried to detect the effect of triptolide on the interactions between TFIIIB components. Briefly, the cells transfected with indicated plasmids expressing FLAG- or Myc-tagged proteins were lysed with RIPA buffer (20 mM Tris-HCl, pH 7.5, 150 mM NaCl, 1 mM EDTA, 1% NP-40) with freshly-added complete protease inhibitor cocktail (Roche Applied Science, Indianapolis, IN). Cell lysates were precipitated with FLAG beads (Sigma) at 4 °C overnight. After washing 3 times with lysis buffer, immunocomplexes were boiled directly in loading buffer and subjected to sodium dodecyl sulfate polyacrylamide gel electrophoresis (SDS-PAGE).

### Fluorescence resonance energy transfer (FRET)

FRET analysis was carried out to detect the effect of triptolide on the interactions between TFIIIB components. FRET was performed as described previously [21]. Plasmids expressing yellow fluorescent protein (YFP)- and cyan fluorescent protein (CFP)- tagged proteins were transfected into RKO cells. Twenty-four hours after transfection, cells were examined with the *Varioskan*Flash (Thermo Fisher Scientific). The wavelength of 458 nm was used for excitation. An emission wavelength of 470–500 nm was used for CFP, and an emission wavelength of 520–550 nm was used for YFP. The ratio of YFP intensity/CFP intensity was used for calculating FRET efficiency. Data from 5 independent experiments were subjected to statistical analysis.

### Human samples

To detect the expression of Pol III products in human CRC samples, 10 paired fresh CRC cancer, and adjacent non-cancer tissues were collected from the tissue bank of the Second Affiliated Hospital of Zhejiang University School of Medicine [22]. The details regarding the samples were disclosed in the Additional file 1: Table S2. A similar amount of starting material was used, including tissue weight, amount of RNA used for reverse transcription, etc. Ethics approval for these studies was obtained from the Ethics Committee of Zhejiang University School of Medicine (#2018–021, 2018 updated) [22]. All procedures performed in this study involving human participants were in accordance with the 1964 Helsinki declaration and its later amendments or comparable ethical standards.

### Statistical analysis

The experiments were repeated at least three times and data were presented as mean ± SD. For the data following normal distribution, statistical significance between two groups was determined with the Student’s *t*-test. Statistical significance among more than 3 groups was determined with one-way analysis of variance (ANOVA). For the CRC tissue samples that do not follow normal distribution, statistical significance between two groups was determined with the Mann-Whitney test. All comparisons were two-tailed and *P* < 0.05 was considered significant.

## Results

### Triptolide attenuates cancer development in AOM/DSS mice

**Fig. 1:**
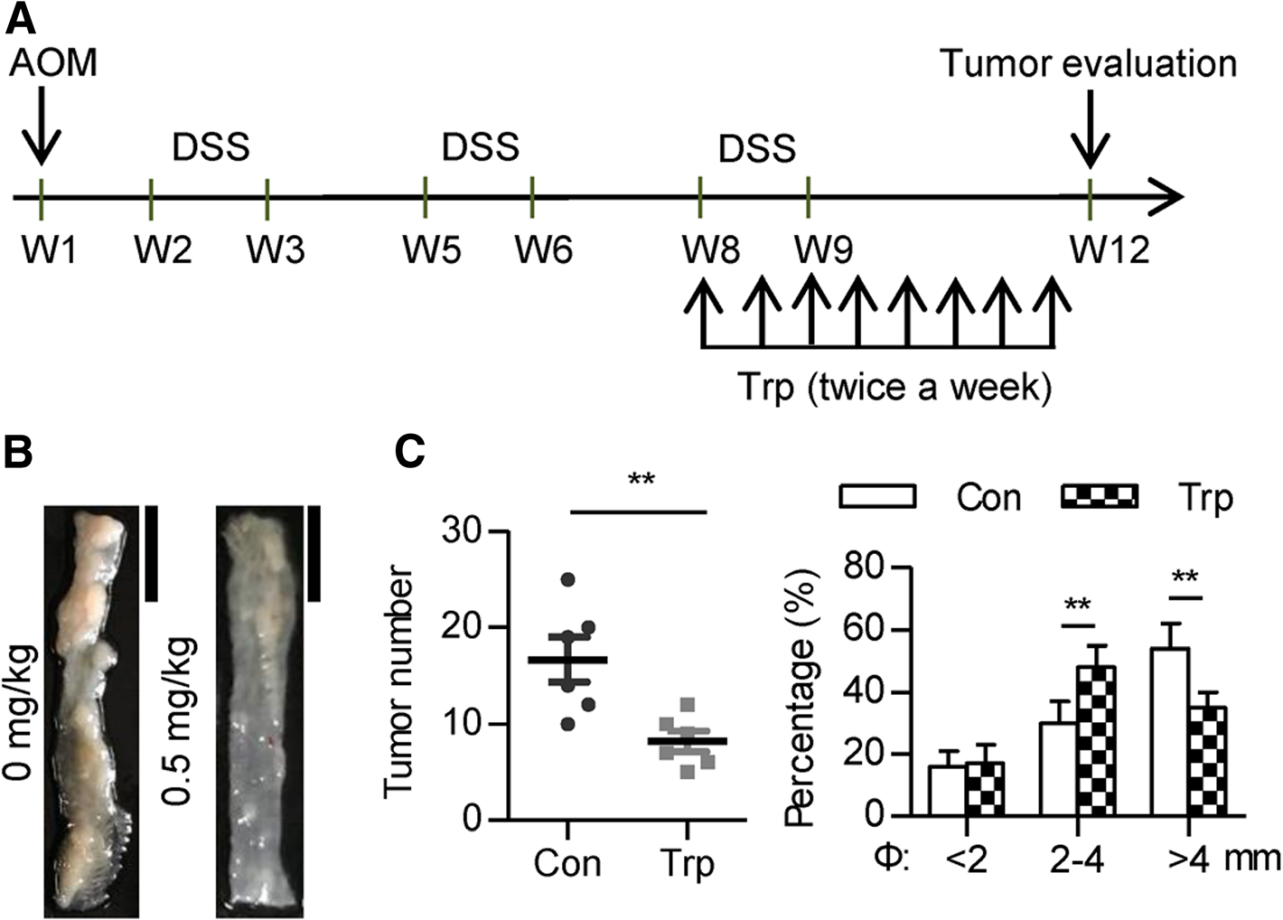
Triptolide attenuates tumor development in AOM/DSS mice. **a** The workflow of the triptolide treatment in AOM/DSS-induced colitis-associated cancer (CAC) model. **b** Representative colons from control or triptolide-treated mice on day 80 of AOM/DSS induction. Scale bar: 1 cm. **c** Colon tumor number and size distribution from control or triptolide-treated. ** *P* < 0.01

To evaluate the therapeutic effect of triptolide in the real context of colorectal cancer development, we firstly applied carcinogen (AOM/DSS)-induced mouse CRC model. Eight-week-old female C57BL/6 J mice were subjected to AOM/DSS induction. After tumor formation, mice were intraperitoneally administrated with either 0.5 mg/kg triptolide in DMSO or DMSO only (control group) twice a week (Fig. 1a). Consistent with the previous study [23], triptolide treatment at this concentration did not induce significant liver toxicity (Additional file 1: Figure S1). DSS water consumption was similar between the two groups. Triptolide treatment at this concentration did not change DSS-induced weight loss compared with the control group. Twelve weeks after tumor induction, the mice were euthanized and the colon was analyzed for tumor number and size. Data showed that triptolide treatment significantly decreased the total tumor burden (Fig. 1b, c). Moreover, the percentage of larger (> 4 mm) tumors decreased while the percentage of middle size (2–4 mm) tumors increased in Trp group, indicating that triptolide decreases the size of AOM/DSS-induced tumor (Fig. 1c).

### Triptolide inhibits tumorigenesis in Apc^Min/+^ mice and represses organoid growth

**Fig. 2:**
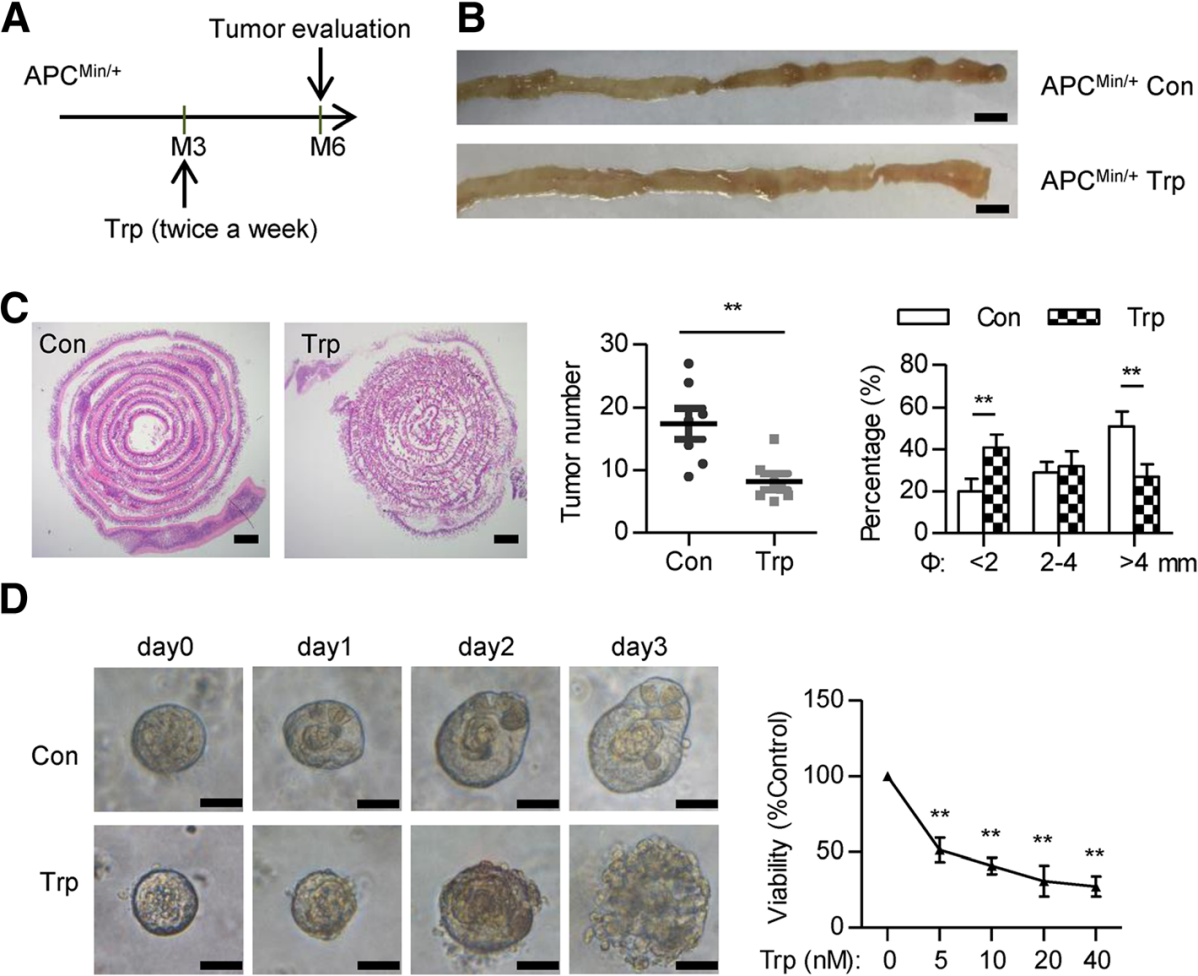
Triptolide inhibits tumorigenesis in Apc^Min/+^ mice and disrupts organoid growth. **a** The workflow of the triptolide treatment in the Apc^Min/+^ mouse model. **b** The small intestines from 6-month-old Apc^Min/+^ mice treated with or without triptolide for 3 months. Scale bar: 1 cm. **c** H&E staining of the small intestines from 6-month-old Apc^Min/+^ mice treated with or without triptolide for 3 months. Scale bar: 1 mm. Tumor number and size distribution from Apc^Min/+^ mice treated with or without triptolide were analyzed. ** *P* < 0.01. **d** Organoids isolated from Apc^Min/+^ mice were treated with or without 5 nM of triptolide and pictures were taken under a microscope. Scale bar: 25 μm. Organoids were treated with different concentration of triptolide for 72 h and cell viability (ATP level) was measured. ** *P* < 0.01

To further confirm the inhibitory effect of triptolide on colorectal cancer development, we used Apc^Min/+^ mouse model, carrying a truncation mutation at the tumor suppressor gene *Apc*, which mainly induces adenoma formation in the small intestine. We treated 3-month-old Apc^Min/+^ mice with triptolide for 3 months and evaluated tumor number and size (Fig. 2a). Weight gain was comparable between groups. Triptolide treatment significantly reduced both tumor burden and tumor size (Fig. 2b, c). These results suggested that upon loss of APC, triptolide reduces tumor formation of small intestinal tumors.

Self-renewal of the intestinal epithelium is driven by Lgr5 stem cells located in crypts [24]. We, therefore, evaluated the effect of triptolide on the growth of organoids from Apc^Min/+^ mice. Data showed that 72 h of triptolide treatment at the concentration of 5 nM induced organoid disruption. We next quantified organoids viability through measurements of ATP levels. Data showed that triptolide reduces organoids viability in a dose-dependent manner (Fig. 2d).

### Triptolide inhibits colorectal cancer cell growth and induces apoptosis

**Fig. 3:**
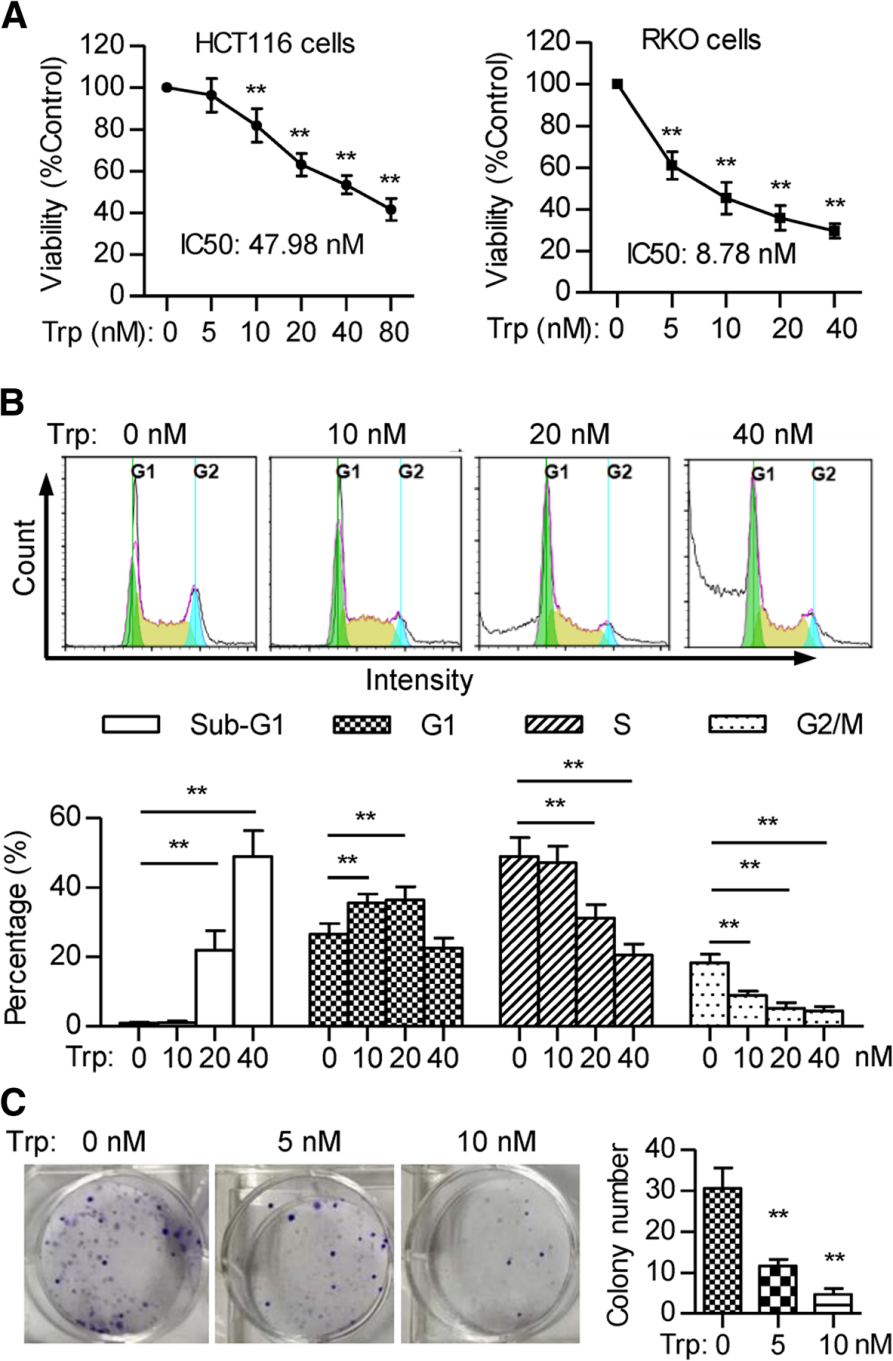
Triptolide inhibits colorectal cancer cell growth and induces apoptosis. **a** HCT116 and RKO cells were incubated with triptolide at different concentrations for 48 h, and cell viability was determined using cell counting kit-8 (CCK-8). ** *P* < 0.01. **b** RKO cells were incubated with triptolide at different concentrations for 24 h and the cell cycle was determined. Cell percentage at G0/G1, S, G2, and sub-G1 phases was calculated and analyzed. **c** Colony formation assay in RKO cells treated with different concentration of triptolide. All the data are presented as the mean ± SD of at least three independent experiments. *P < 0.05, **P < 0.01

Further, we evaluated the effect of different doses of triptolide on the viability of colon cancer cells. Data showed that triptolide decreased the viability of both HCT116 and RKO colon cancer cells in a dose-dependent fashion (Fig. 3a). To identify the cellular events that inhibit RKO cell proliferation, we used propidium iodide staining to analyze cell cycle. Data showed that a lower concentration of triptolide (10 nM) treatment induced cells accumulation at G0/G1 cycle, while a higher concentration of triptolide (> 20 nM) increased the sub-G1 population, which are apoptotic cells (Fig. 3b). Triptolide mainly induced G2 cycle arrest and cell apoptosis in HCT116 cells (Additional file 1: Figure S2). In addition, triptolide treatment significantly decreased colony formation of RKO cells (Fig. 3c).

### Triptolide inhibits pol III transcription

**Fig. 4:**
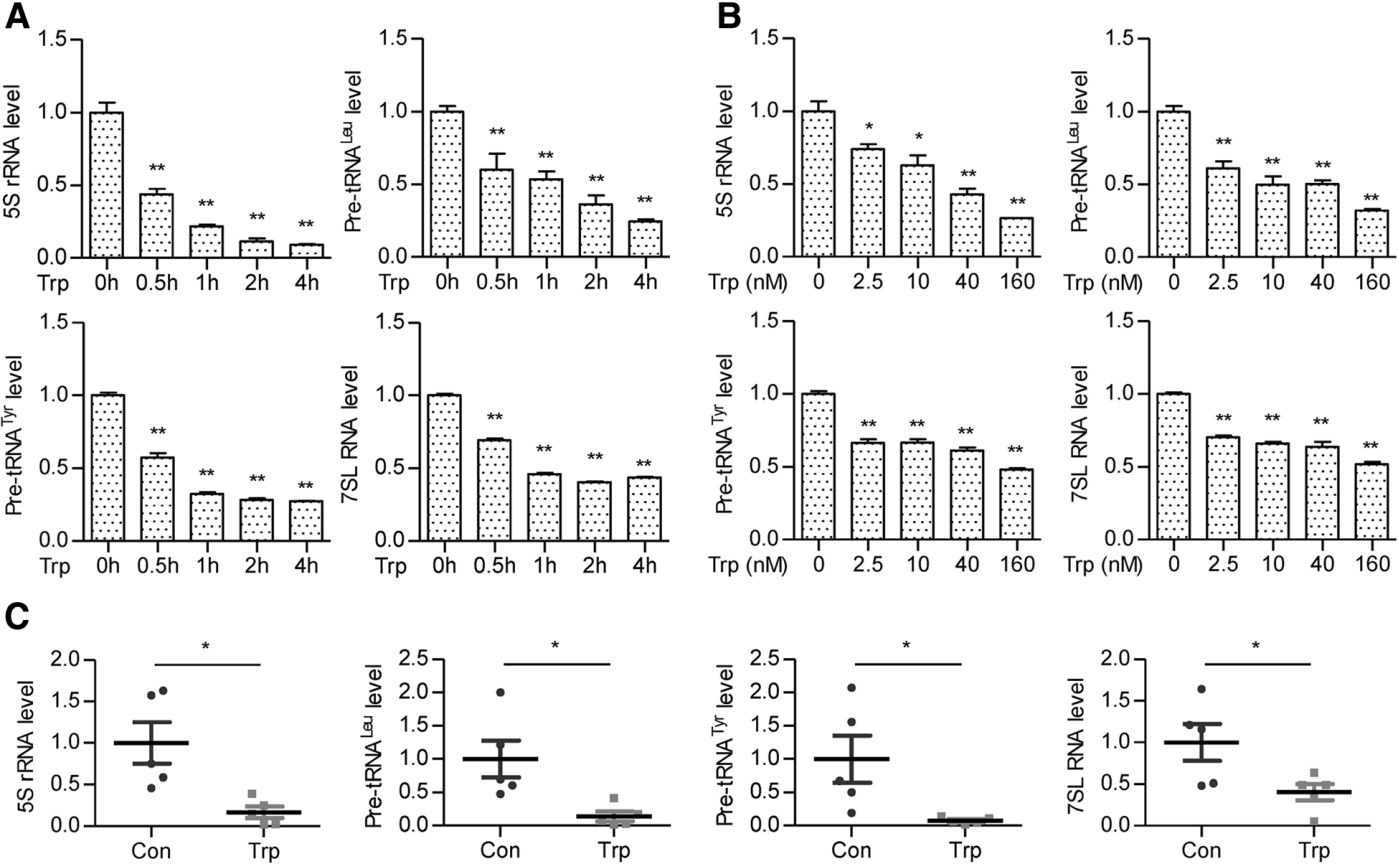
Triptolide inhibits Pol III transcription. **a** RKO cells were incubated with 40 nM triptolide for the indicated time. The mRNA level of 5S rRNA, pre-tRNA^Leu^, pre-tRNA^Tyr^, and 7SL RNA was measured by real-time qPCR. **b** RKO cells were incubated with triptolide at the indicated concentration for 0.5 h. The mRNA level of 5S rRNA, pre-tRNA^Leu^, pre-tRNA^Tyr^, and 7SL RNA was measured by real-time qPCR. **c** The mRNA level of 5S rRNA, pre-tRNA^Leu^, pre-tRNA^Tyr^, and 7SL RNA from triptolide-treated and untreated tumors from AOM/DSS mice was measured by real-time qPCR. All the data are presented as the mean ± SD of at least three independent experiments. *P < 0.05, **P < 0.01

Triptolide could inhibit both Pol I and Pol II transcription. Pol III products, including tRNAs, 5S rRNA, control mRNA translation efficiency and growth capacity of cells [14, 15]. We hypothesize that triptolide also inhibits Pol III transcription. To our surprise, 40 nM of triptolide treatment for only 0.5 h dramatically reduced the expression of Pol III products including 5S rRNA, pre-tRNA^Leu^, pre-tRNA^Tyr^, and 7SL RNA. The expression level of these genes further decreased with time extends (Fig. 4a). Triptolide inhibited Pol III transcription in a dose-dependent manner, and the inhibitory effect could be observed at the concentration of 2.5 nM (Fig. 4b). The inhibition of triptolide on Pol III was also observed in HCT116 cells (Additional file 1: Figure S3). Moreover, tumors from triptolide-treated AOM/DSS mouse showed reduced Pol III transcription compared with the control group, confirming the inhibitory effect of triptolide on Pol III transcription (Fig. 4c).

### Triptolide inhibits mRNA translation efficiency

**Fig. 5:**
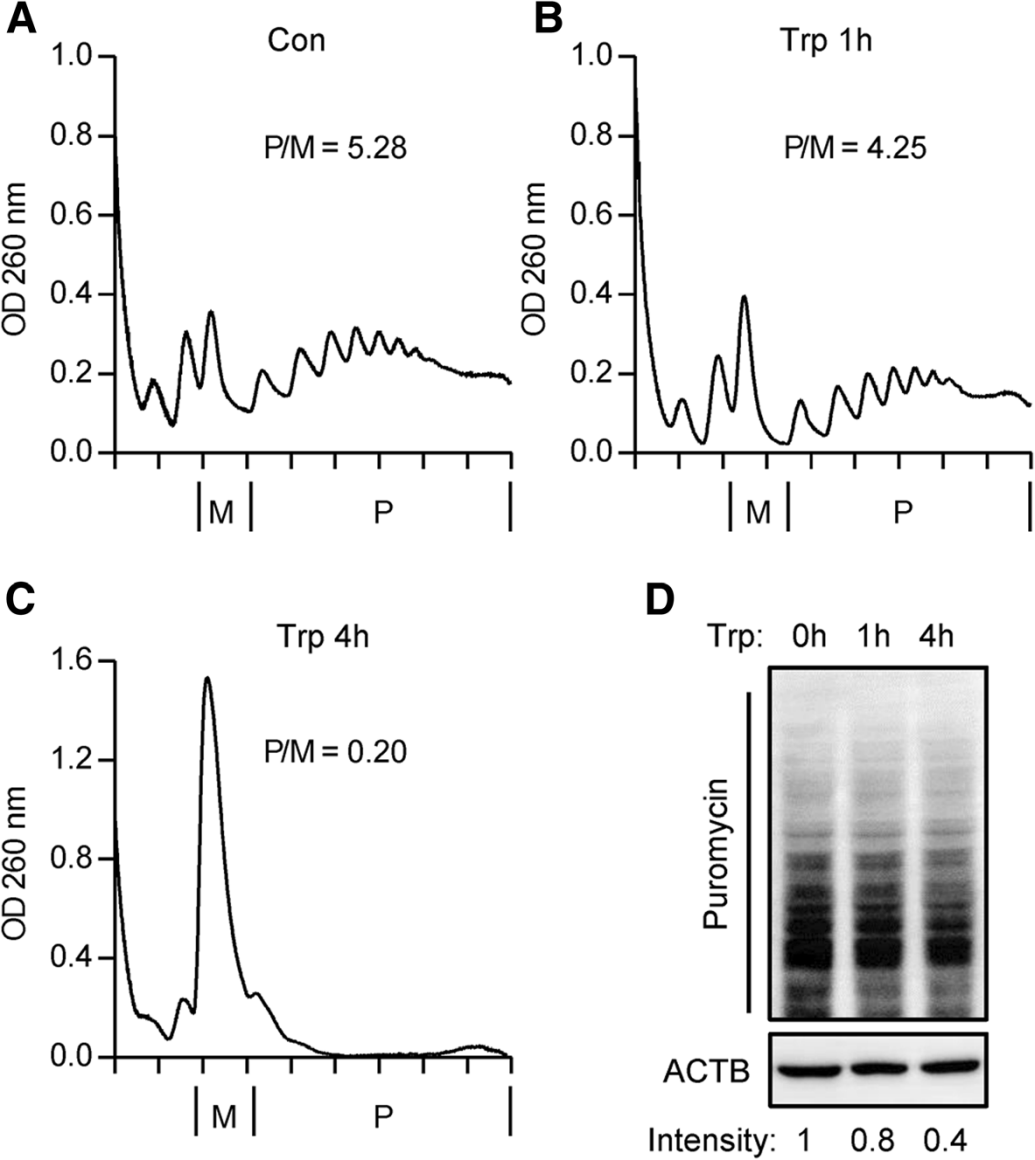
Triptolide inhibits mRNA translation efficiency. **a**, **b**, **c** RKO cells were treated with 40 nM triptolide for the indicated time. Polysome profiling was carried out. **d** RKO cells were treated with 40 nM triptolide for the indicated time and the newly synthesized peptides were detected by puromycin labeling

To detect whether reduced Pol III transcription affects mRNA translation efficiency, we performed polysome profiling. 1 h of triptolide treatment at the concentration of 40 nM slightly reduced the polysome/monosome (P/M) ratio, while 4 h of triptolide treatment dramatically decreased P/M ratio, indicating the reduced translation efficiency (Fig. 5a, b, c). As an independent validation, we examined the synthesis of endogenous proteins using puromycin labeling [25]. In agreement with polysome profiling, the total levels of puromycin-labeled nascent chains were significantly decreased after 4 h of triptolide treatment (Fig. 5d).

### Triptolide disrupts TFIIIB formation at pol III promoters

**Fig. 6:**
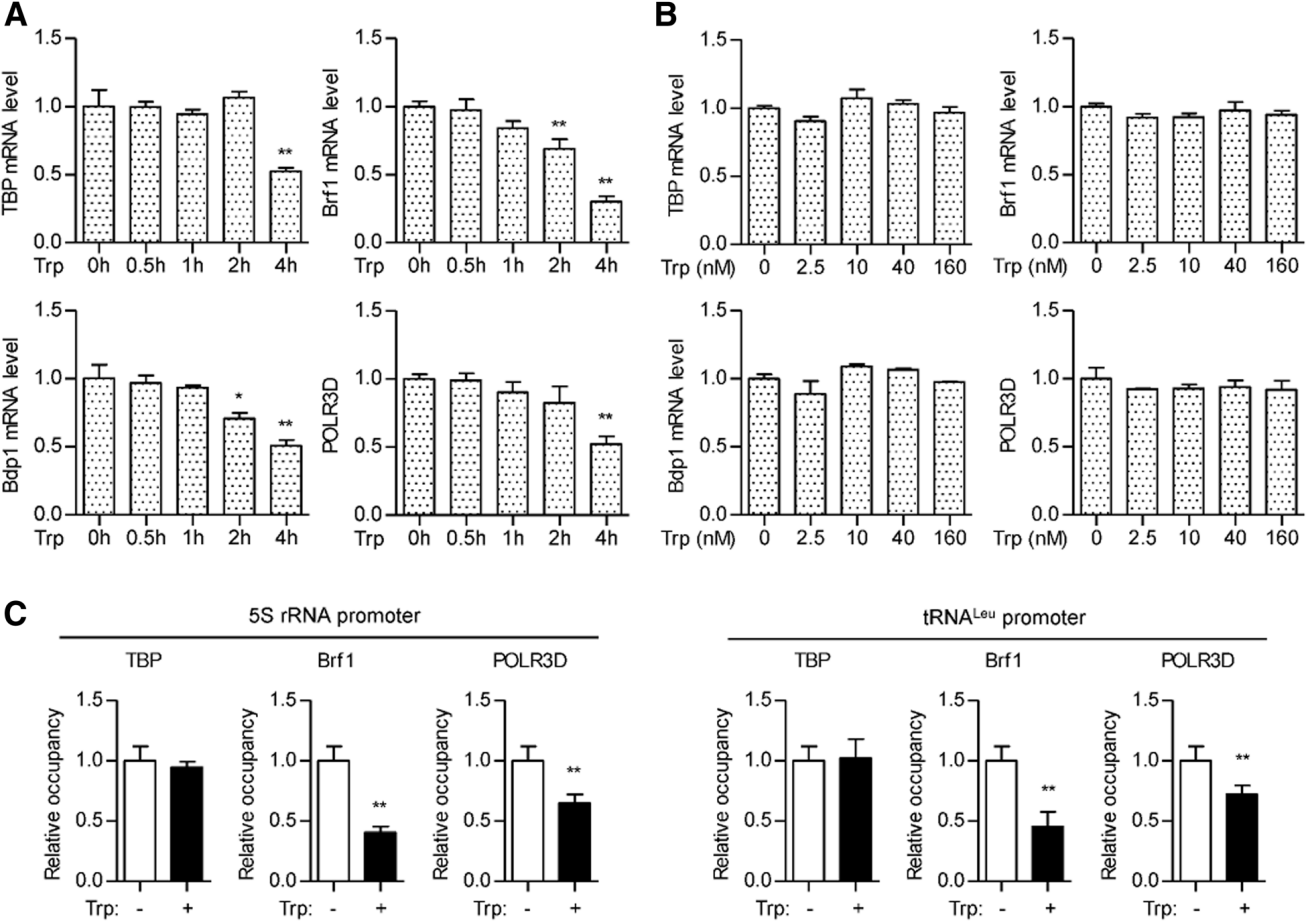
Triptolide represses TFIIIB formation at Pol III promoters. **a** RKO cells were incubated with 40 nM triptolide for the indicated time. The mRNA level of TBP, Brf1, Bdp1, and POLR3D was measured by real-time qPCR. **b** RKO cells were incubated with triptolide at the indicated concentration for 0.5 h. The mRNA level of TBP, Brf1, Bdp1, and POLR3D was measured by real-time qPCR. **c** RKO cells expressing FLAG-tagged TBP, Brf1, or POLR3D were treated with or without 40 nM triptolide for 0.5 h. Chromatin immunoprecipitation was carried out using FLAG antibody and the immunoprecipitates were subjected to qPCR

The principal transcription initiation factor of Pol III is TFIIIB, a complex consisting of TBP, Brf1, and Bdp1. To explore the potential mechanism of reduced Pol III transcription, we detected the expression of TBP, Brf1, Bdp1, and POLR3D, one subunit of Pol III. We observed reduced expression of these genes after 2 h of triptolide treatment, which is later than decreased Pol III products. 0.5 h of triptolide treatment did not affect the expression level of these genes even at very high concentration (160 nM) (Fig. 6a, b). Therefore, triptolide-inhibited Pol III transcription was not due to the decreased expression of Pol III machinery.

During the initiation of Pol III transcription, TBP mediates the binding of TFIIIB with promoter DNA, while Brf1 holds together the trimeric TBP-Brf1-Bdp1 complex. Thereafter, Brf1 and Bdp1 work together to recruit Pol III to form a transcriptionally active preinitiation complex (PIC) [26, 27]. To determine the effect of triptolide on PIC formation, we labeled each subunit with the FLAG tag and performed ChIP experiments. Data showed that 0.5 h of triptolide treatment reduced the recruitment of POLR3D at promoters of 5S rRNA and pre-tRNA^Leu^, which is consistent with the reduced Pol III transcription. Triptolide did not affect the binding of TBP with the promoter DNA, but significantly reduced the recruitment of Brf1 (Fig. 6c), suggesting the repressed TFIIIB formation at these promoters.

### Triptolide blocks the interaction between TBP and Brf1

**Fig. 7:**
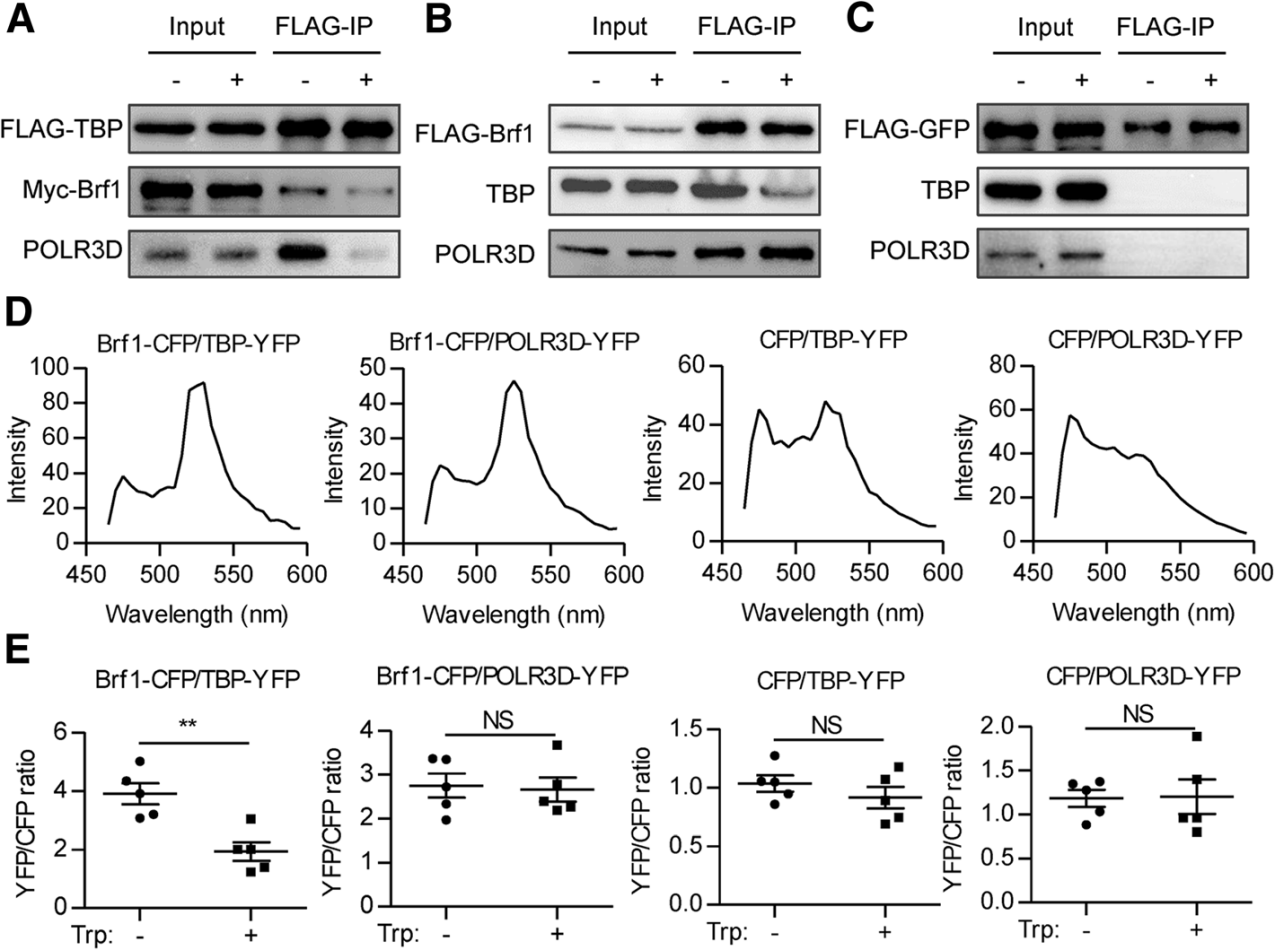
Triptolide disrupts the interaction between TBP and Brf1. **a** RKO cells expressing FLAG-TBP, Myc-Brf1 were immunoprecipitated with FLAG antibody. The immunoprecipitates were detected with the indicated antibodies. **b** RKO cells expressing FLAG-Brf1 were immunoprecipitated with FLAG antibody. The immunoprecipitates were detected with the indicated antibodies. **c** RKO cells expressing FLAG-GFP were immunoprecipitated with FLAG antibody. The immunoprecipitates were detected with the indicated antibodies. **d** RKO cells expressing the indicated proteins were excited at the wavelength of 458 nm and emission signal between 460 nm to 600 nm was detected at the 10 nm interval. **e** RKO cells expressing the indicated proteins were treated with 40 nM triptolide for 0.5 h. Cells were excited at the wavelength of 458 nm and YFP (470-500 nm)/CFP (520-550 nm) ratio was calculated

Data above prompted us to detect the interaction between TFIIIB components after triptolide treatment. We first carried out the Co-IP experiments. In agreement with the ChIP results, triptolide reduced the binding of TBP with Brf1 and POLR3D. However, triptolide treatment did not affect the interaction between Brf1 and POLR3D (Fig. 7a, b). These data indicated that triptolide targets TBP/Brf1 binding. As a negative control, FLAG-tagged GFP did not interact with either TBP or POLR3D (Fig. 7c).

To further confirm the Co-IP results, we performed FRET analysis. CFP-tagged Brf1 and YFP-tagged TBP were excited with the appropriate laser line (458 nm for CFP or 514 nm for YFP). Emission spectra scanning demonstrated that the emission Brf1-CFP and TBP-YFP peaks at about 475 nm and 525 nm respectively. Cells expressing both Brf1-CFP and TBP-YFP were excited at 458 nm to detect if there is FRET between these two proteins. We observed a decrease in the CFP emission and an increase in the YFP emission, demonstrating that FRET did happen between Brf1-CFP and TBP-YFP. FRET was detected in Brf1-CFP/POLR3D-YFP, but neither in CFP/TBP-YFP nor in CFP/POLR3D-YFP (Fig. 7d).

Taken advantage of YFP/CFP intensity ratio reflecting TBP/Brf1 interaction, we detected the interaction between TBP/Brf1 real-timely after adding triptolide to the medium. The YFP/CFP ratio decreased significantly 30 min after triptolide addition, demonstrating the disruption of TBP/Brf1 binding (Fig. 7e). In line with Co-IP results, triptolide did not affect FRET efficiency between Brf1-CFP and POLR3D-YFP (Fig. 7e).

### Pol III products are upregulated in CRC

**Fig. 8:**
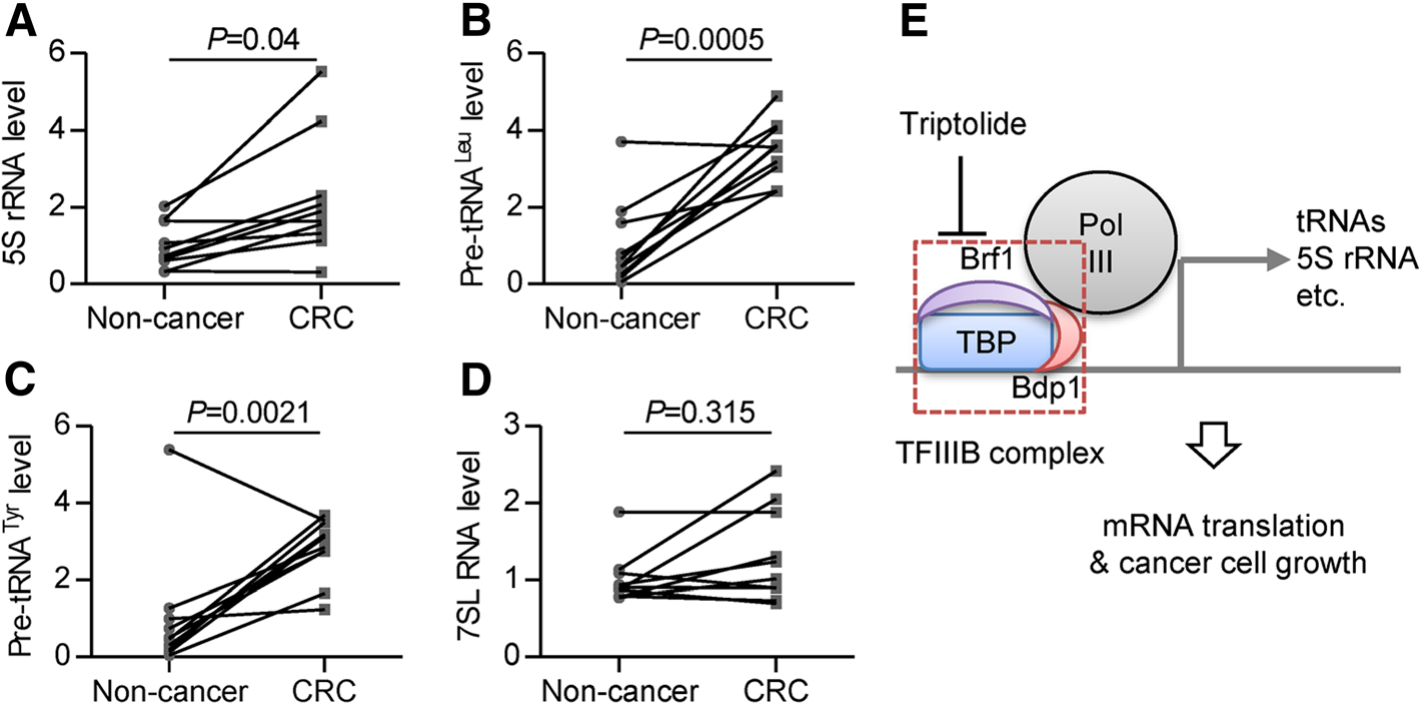
Pol III products are upregulated in CRC. **a**, **b**, **c**, **d** The expression of 5S rRNA (**a**), pre-tRNA^Leu^ (**b**), pre-tRNA^Tyr^ (**c**), and 7SL RNA (**d**) from CRC tissues or adjacent non-cancer tissues was measured by real-time qPCR. The expression level was calculated as fold change with ACTB as endogenous control and normalized to the mean of non-cancerous group values. Dots indicate individual values. (**e**) The working model for triptolide-inhibited Pol III transcription and cancer cell growth

Given that triptolide targets Pol III in cultured cancer cells and animal intestinal cancer model, we continued to investigate the relationship between Pol III transcription and CRC. We examined Pol III products, including 5S rRNA, pre-tRNA^Leu^, pre-tRNA^Tyr^, and 7SL RNA in 10 paired CRC patient samples. RT-qPCR measurement revealed that the expression of 5S rRNA, pre-tRNA^Leu^, pre-tRNA^Tyr^ was significantly increased in CRC tissues comparing to the adjacent non-cancer ones (Fig. 8), implying an oncogenic activity of Pol III products in CRC development.

## Discussion

In the current study, we demonstrate that triptolide, a diterpene triepoxide from a Chinese herb Trypterigium wilfordii, inhibits colorectal carcinogenesis in both Apc^Min/+^ and AOM/DSS mouse models. Our study strongly indicates that triptolide and triptolide-like drugs are promising colorectal cancer therapies. Importantly, we demonstrate that triptolide inhibits Pol III-dependent 5S rRNA and tRNAs transcription. To our knowledge, this is the first report demonstrating that triptolide inhibits Pol III transcription.

Triptolide has been shown to be effective against colon cancer cell growth [28]. However, most of these studies are based on in vitro cell models or xenograft models, which do not reflect the real context of colorectal cancer tumorigenesis. Our study, for the first time, demonstrated the anticancer effect of triptolide on colorectal cancer formation in both Apc^Min/+^ and AOM/DSS mice. Moreover, using cultured 3D organoids, we demonstrated that triptolide at nanomolar concentration could inhibit organoid growth from Apc^Min/+^ mouse. Since the growth of Apc^Min/+^ organoids reflects the self-renewal activity of intestinal stem cells, our data suggested a role of triptolide in inhibiting colorectal cancer stem cell function. Of note, our data showed that triptolide can also inhibit the growth of normal intestinal organoids at higher concentration compared with the organoids from Apc^Min/+^ mice (Additional file 1: Figure S4). We proposed that, like most of the chemical drugs, triptolide does not have cell type selectivity. However, the cancer cells have a higher level of Pol III transcription, making them more sensitive to triptolide treatment.

Although the precise molecular targets remain elusive, recent studies in cancer cells have revealed that RNA polymerase may be an important target of triptolide. McCallum et al. showed that triptolide inhibits de novo total RNA transcription [9]. Numerous evidence has demonstrated that triptolide inhibits Pol II transcription initiation [10, 11, 29]. In addition, triptolide could inhibit Wnt signaling in non-small cell lung carcinoma via epigenetic modifications to histone H3, indicating an inhibitory effect of triptolide on Pol II expression at the epigenetic level [30]. Leuenroth and Crews demonstrated that triptolide induced disappearance of normal nucleolar structure [12], which accounts for the reduced RNA Pol I transcription and subsequent ribosome biogenesis. The Pol III products including tRNAs and 5S rRNA are translation machinery, which controls mRNA translation efficiency [14, 15]. Therefore, it is reasonable to propose that Pol III transcription should be inhibited to coordinate with reduced Pol I and Pol II transcription. Indeed, triptolide treatment reduced the expression level of 5S rRNA and tRNAs in cultured colorectal cancer cells as well as in mouse colorectal cancer tissue. Accordingly, mRNA translation efficiency was dramatically inhibited after triptolide treatment. Interestingly, our results showed that 0.5 h of triptolide treatment did not affect the expression of TFIIIB components but blocked the formation of TFIIIB, suggesting a direct effect of triptolide on Pol III transcription machinery other than the side effect from Pol II inhibition. We observed that a long time of triptolide treatment inhibited the expression of TFIIIB components due to the inhibition of Pol II. Therefore, triptolide might inhibit Pol III transcription through both direct and indirect mechanisms.

TFIIIB plays an important role in recruiting Pol III to its target genes, making it an ideal target for regulation. By targeting TFIIIB, oncogenic proteins such as Ras, c-Jun, and c-Myc stimulate RNA Pol III transcription [31, 32], whereas tumor suppressors such as pRb, p53, PTEN, and Maf1 repress Pol III transcription [14, 32]. The formation of TFIIIB complex at the promoter region requires multiple contacts between components of the basal transcription apparatus. First, TBP is recruited to the promoter of Pol III genes. Then, Brf1 and Bdp1 join to form TFIIIB complex at the class III gene promoters. After that, TFIIIB could recruit Pol III and initiate transcription. Our ChIP experiments showed that the loading of TBP to the promoter of 5S rRNA and tRNAs did not change under 0.5 h of triptolide treatment, while the loading of Brf1 and POLR3D was repressed (Fig. 6c). Co-IP and FRET experiments demonstrated that triptolide blocks TBP/Brf1 interaction without affecting Brf1/POLR3D interaction (Fig. 7). Based on these findings, we proposed a model that triptolide directly disrupts TFIIIB formation to inhibit Pol III transcription (Fig. 8e). Since most of Pol III products are the essential components of translation machinery, Pol III transcription is upregulated (Fig. 8) in cancer cells to satisfy the increased anabolic demands associated with cell growth. Due to its inhibitory effect on Pol III transcription, we envision that triptolide might serve as an effective drug candidate for CRC therapy.

## Conclusions

In conclusion, besides inhibiting the transcription of Pol II and Pol I, triptolide also inhibits Pol III transcription by disrupting TFIIIB formation, leading to the suppression of mRNA translation (Fig. 8). The capability of triptolide to induce colorectal cancer cell growth arrest both in vitro and in vivo makes it a potential therapeutic drug candidate for CRC.

## Additional file

Additional file 1: **Table S1** Primers used in this study. **Table S2** Details of the CRC samples used in this study. **Figure S1** The effect of triptolide on liver function. **Figure S2** Triptolide induces G2 cycle arrest and apoptosis in HCT116 cells. **Figure S3** Triptolide inhibits Pol III transcription in HCT116 cells. **Figure S4** The effect of triptolide on the growth of normal organoids. (DOCX 336 kb)

## Abbreviations

AOM: Azoxymethane
Apc: Adenomatous polyposis coli
Bdp1: B double prime 1
Brf1: B-related factor 1
CAC: Colitis-associated cancer
CFP: Cyan fluorescent protein
ChIP: Chromatin immunoprecipitation
Co-IP: Co-immunoprecipitation
CRC: Colorectal cancer
DMSO: Dimethyl sulfoxide
DSS: Dextran sodium sulfate
EDTA: Ethylenediaminetetraacetic acid
FRET: Fluorescence resonance energy transfer
PCR: Polymerase chain reaction
Pol III: RNA polymerase III
SDS-PAGE: Sodium dodecyl sulfate polyacrylamide gel electrophoresis
TBP: TATA box-binding protein
YFP: Yellow fluorescent protein
