# Supplementary material for: Inhibition of RNA polymerase III transcription by Triptolide attenuates colorectal tumorigenesis
Source: J Exp Clin Cancer Res. 2019 May 23;38:217. doi: 10.1186/s13046-019-1232-x (PMC6533717; doi:10.1186/s13046-019-1232-x)
Supplement: Supplementary file 1 — Table S1 Primers used in this study. Table S2 Details of the CRC samples used in this study. Figure S1 The effect of triptolide on liver function. Figure S2 Triptolide induces G2 cycle arrest and apoptosis in HCT116 cells. Figure S3 Triptolide inhibits Pol III transcription in HCT116 cells. Figure S4 The effect of triptolide on the growth of normal organoids. (DOCX 336 kb) [file 13046_2019_1232_MOESM1_ESM.docx]

**Inhibition of RNA Polymerase III Transcription by Triptolide Attenuates** **Colorectal** **Tumorigenesis**

Xia Liang^1,#^, Renxiang Xie^2,#^, Jinfeng Su^1,#^, Bingqi Ye^2^, Saisai Wei^3^, Zhibing Liang^1^, Rongpan Bai^2^, Zhanghui Chen^4^, Zhongxiang Li^1,*^, Xiangwei Gao^2,*^.

1, Medical Research Institute, Baoan Maternal and Child Health Hospital, Jinan University, Shenzhen, 518102, China.

2, Department of Public Health, Zhejiang University School of Medicine, Hangzhou, 310058, China.

3, Sir Run-Run Shaw Hospital, Zhejiang University School of Medicine, Hangzhou, 310058, China.

4, Affiliated Central People’s Hospital of Zhanjiang, Guangdong Medical University, Zhanjiang, 524045, China.

# These authors contributed equally.

* Correspondence should be addressed to: Xiangwei Gao, Ph.D., [xiangweigao@zju.edu.cn](mailto:xiangweigao@zju.edu.cn); or Zhongxiang Li, Ph.D., [zhongxiangli@gmail.com](mailto:zhongxiangli@gmail.com).

**Running Title:** Triptolide targets Pol III.

**SUPPLEMENTARY INFORMATION**

**Supplementary Tables**

**Supplementary Figures**

**Supplementary table 1. Primers used in this study.**

| **Gene name** | **Primer name** | **Sequence** |
| --- | --- | --- |
| Human *β-actin* | H-ACTB-F | 5’-CACGATGGAGGGGCCGGACTCATC-3’ |
|  | H-ACTB-R | 5’-TAAAGACCTCTATGCCAACACAGT-3’ |
| Human *5S rRNA* | H-5S-F | 5’-GGCCATACCACCCTGAACGC-3’ |
|  | H-5S-R | 5’-CAGCACCCGGTATTCCCAGG-3’ |
| Human *Leu tRNA* | H-Leu-F | 5’-CAGGATGGCCGAGCGGT-3’ |
|  | H-Leu-R | 5’-TCAGGAGTGGGATTCGAAC-3’ |
| Human *Tyr tRNA* | H-Try-F | 5’-CCTTCGATAGCTCAGCTGGT-3’ |
|  | H-Try-R | 5’-CGACCTAAGGATGTCCACAAAT-3’ |
| Human *7SL RNA* | H-7SL-F | 5’-CAAAACTCCCGTGCTGATCA-3’ |
|  | H-7SL-R | 5’-GGCTGGAGTGCAGTGGCTAT-3’ |
| Human *Brf1* | H-Brf1-F | 5’-TCTCAGACTCCATCCGCGAAT-3’ |
|  | H-Brf1-R | 5’-GCGTTCTCCCTCATCCACA-3’ |
| Human *TBP* | H-TBP-F | 5’-CCACTCACAGACTCTCACAAC-3’ |
|  | H-TBP-R | 5’-CTGCGGTACAATCCCAGAACT-3’ |
| Human *Bdp1* | H-Bdp1-F | 5’-TATTTGAGCGCGGTTCTACAAC-3’ |
|  | H-Bdp1-R | 5’-AAAGTCAGTTCCTACCATGCTG-3’ |
| Human *POLR3D* | H-POLR3D-F | 5’-GGCCGTCCAGAAGTGATCC-3’ |
|  | H-POLR3D-R | 5’-CATCCACTGTCTTATCCCAGTTC-3’ |
| Mouse *β-actin* | Mβ-actin-F | 5’-GCAGATGTGGATCAGCAAGC-3’ |
|  | Mβ-actin-R | 5’-AGCTCAGTAACAGTCCGCC-3’ |
| Mouse *5S rRNA* | H-5S-F | 5’-GGCCATACCACCCTGAACGC-3’ |
|  | H-5S-R | 5’-CAGCACCCGGTATTCCCAGG-3’ |
| Mouse *Leu tRNA* | H-Leu-F | 5’-GTCAGGATGGCCGAGTGGTCTAAG-3’ |
|  | H-Leu-R | 5’-CCACGCCTCCATACGGAGAACCAGAAGACCC-3’ |
| Mouse *Tyr tRNA* | H-Try-F | 5’-CCTTCGATAGCTCAGCTGGTAGAGCGGAGG-3’ |
|  | H-Try-R | 5’-CGGAATTGAACCAGCGACCTAAGGATGTCC-3’ |
| Mouse *7SL RNA* | H-7SL-F | 5’-GTGTCCGCACTAAGTTCGG-3’ |
|  | H-7SL-R | 5’-TATTCACAGGCGCGATCC-3’ |
| Human *Brf1-FLAG* | H-Brf1F-F | ATCGAAGCTTGCCACCATGACGGGCCGCGTGTG |
|  | H-Brf1F-R | TAGCGAATTCGCGTAGCCGTCGTCCTCATCG |
| Human *TBP-FLAG* | H-TBPF-F | ATCGAAGCTTGCCACCATGGATCAGAACAACAGCCTG |
|  | H-TBPF-R | TAGCGAATTCGCCGTCGTCTTCCTGAATCC |
| Human *POLR3D-FLAG* | H-POLR3DF-F | ATCGAAGCTTGCCACCATGTCGGAAGGAAACGCCG |
|  | H- POLR3DF -R | TAGCGAATTCGCCCGGTGTTTGTGATCCAAG |

**Supplementary table 2. Details of the CRC samples used in this study.**

| Patient number | Gender | History of chemotherapy | Type of lesion | Primary lesion site | Lymph node metastasis | Distant metastasis | Stage (AJCC) |
| --- | --- | --- | --- | --- | --- | --- | --- |
| 1 | Male | None | Adenocarcinoma | Colon | None | None | II |
| 2 | Female | None | Adenocarcinoma | Colon | None | None | II |
| 3 | Male | None | Adenocarcinoma | Colon | Yes | None | III |
| 4 | Female | None | Adenocarcinoma | Colon | Yes | Yes | IV |
| 5 | Female | None | Adenocarcinoma | Colon | Yes | None | III |
| 6 | Male | None | Adenocarcinoma | Colon | Yes | None | III |
| 7 | Male | None | Adenocarcinoma | Colon | Yes | Yes | IV |
| 8 | Male | None | Adenocarcinoma | Colon | None | None | II |
| 9 | Male | None | Adenocarcinoma | Colon | None | None | II |
| 10 | Female | None | Adenocarcinoma | Colon | None | None | II |

**Supplementary Figure 1. The effect of triptolide on liver function.**


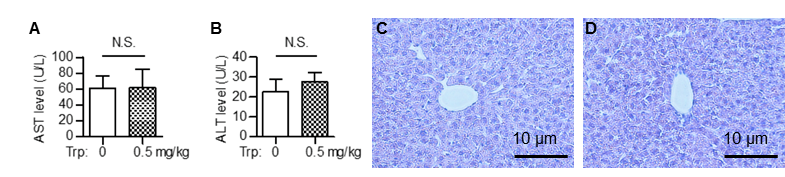


(A, B) Mice treated with or without triptolide (0.5 mg/kg) for 1 week and the blood level of AST (A) and ALT (B) was determined. (C, D) HE staining pictures of liver tissue sections from mouse treated without (C) or with (D) triptolide (100×).

**Supplementary Figure 2. Triptolide induces G2 cycle arrest and apoptosis in HCT116 cells.**


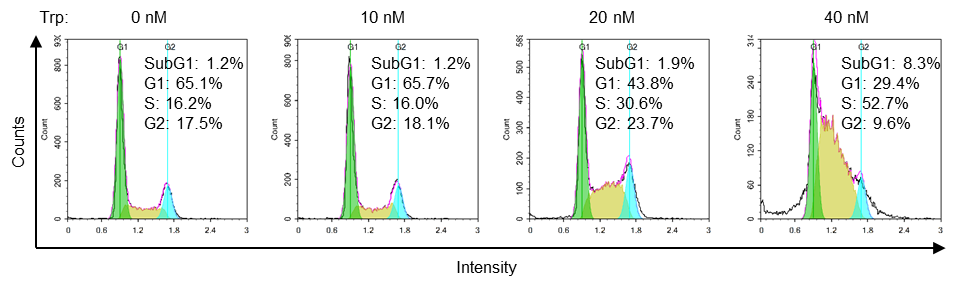


HCT116 cells were incubated with triptolide at different concentrations for 24 h and the cell cycle was determined. Cell percentage at G0/G1, S, G2, and sub-G1 phases was calculated and analyzed.

**Supplementary Figure 3. Triptolide inhibits Pol III transcription in HCT116 cells.**

**
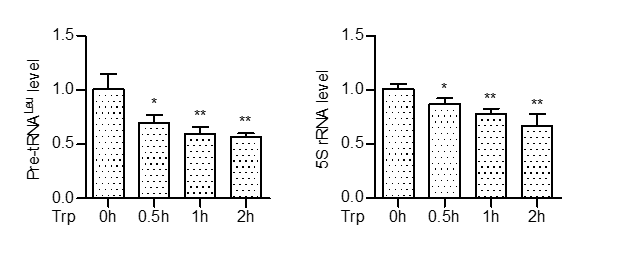
**

HCT116 cells were incubated with 40 nM triptolide for the indicated time. The mRNA level of 5S rRNA and pre-tRNA^Leu^ was measured by real-time qPCR.

**Supplementary Figure 4. The effect of triptolide on the growth of normal organoids.**

**
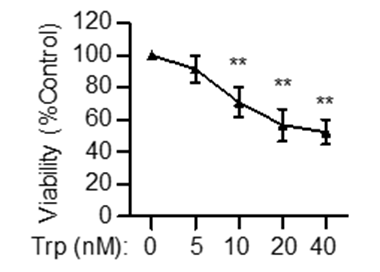
**

Organoids isolated from wild type mouse were treated with different concentration of triptolide for 72 hours and cell viability (ATP level) was measured. ** *P*<0.01.
